# Brain Meta-Transcriptomics from Harbor Seals to Infer the Role of the Microbiome and Virome in a Stranding Event

**DOI:** 10.1371/journal.pone.0143944

**Published:** 2015-12-02

**Authors:** Stephanie M. Rosales, Rebecca Vega Thurber

**Affiliations:** Oregon State University, Dept. of Microbiology, 226 Nash Hall, Corvallis, OR, 97331, United States of America; U.S. Geological Survey, UNITED STATES

## Abstract

Marine diseases are becoming more frequent, and tools for identifying pathogens and disease reservoirs are needed to help prevent and mitigate epizootics. Meta-transcriptomics provides insights into disease etiology by cataloguing and comparing sequences from suspected pathogens. This method is a powerful approach to simultaneously evaluate both the viral and bacterial communities, but few studies have applied this technique in marine systems. In 2009 seven harbor seals, *Phoca vitulina*, stranded along the California coast from a similar brain disease of unknown cause of death (UCD). We evaluated the differences between the virome and microbiome of UCDs and harbor seals with known causes of death. Here we determined that UCD stranded animals had no viruses in their brain tissue. However, in the bacterial community, we identified *Burkholderia* and *Coxiella burnetii* as important pathogens associated with this stranding event. *Burkholderia* were 100% prevalent and ~2.8 log2 fold more abundant in the UCD animals. Further, while *C*. *burnetii* was found in only 35.7% of all samples, it was highly abundant (~94% of the total microbial community) in a single individual. In this harbor seal, *C*. *burnetii* showed high transcription rates of invading and translation genes, implicating it in the pathogenesis of this animal. Based on these data we propose that *Burkholderia* taxa and *C*. *burnetii* are potentially important opportunistic neurotropic pathogens in UCD stranded harbor seals.

## Introduction

Emerging infectious diseases are on the rise in both humans and wildlife. Hence, preemptive pathogen surveillance is necessary to better-forecast disease outbreaks [1,2]. Currently, it is thought that about 61% of emerging human diseases arise from zoonotic pathogens and ~70% of these originate from wildlife [1,3]. Evidently, emerging diseases are likely to be zoonotic, such as the Ebola outbreak of 2013–2014 and the Middle East Respiratory Syndrome Coronavirus (MERS-CoV) of 2012 and 2014 [3–5]. Recent outbreaks like these exemplify the severity and need to evaluate the origins of zoonoses.

### Marine mammal zoonoses

Currently, there are about 15 known zoonotic marine mammal pathogens (reviewed in [6]). For instance, *Mycobacterium tuberculosis*, the bacterial pathogen that causes tuberculosis, was introduced to the Americas via pinnipeds [7]. In addition, Influenza A virus, which poses a global human threat, is present in cetacean and pinniped populations and has been shown to be transmitted from seals to humans [8–10]. Since aquatic mammals are phylogenetically our closest sea relatives they serve as sentinel species for both human and ocean-related health [11]. Thus identifying pathogens in marine mammals may help assuage disease outbreaks and prevent zoonotic transmission [12].

### Marine mammal strandings as an important resource for zoonotic disease surveillance

Marine mammals are susceptible to strandings, which is defined by the Marine Mammal Protection Act as a marine mammal that is dead or alive on the shore or beach. Infectious disease is highly associated with marine mammal stranding events. For instance, in Massachusetts, a survey of 405 stranded pinnipeds and cetaceans concluded that diseases were linked to the largest proportion (37%) of animal deaths [13]. Although there are efforts to examine the roots of some of these stranding events, many go undetermined [14]. More thorough examination of the infectious base of marine mammals mortalities should be conducted, since 44% of stranded marine mammals die from unknown causes [15].

Stranded animals can supply an ideal source of information for the identification of emerging infectious diseases in marine mammal populations. For example, investigations of stranded harbor seals *Phoca vitulina* in 1998 and 2002 concluded that morbillivirus caused the death of 23,000 and 30,000 harbor seals, respectively [16]. Unfortunately, harbor seals have not been the only marine mammals affected by this virus; strandings of pinnipeds and cetaceans has led to the discovery of four new morbillivirus types (PDV, CMV, CDV, and MSMV). The importance of these discoveries is evident in the number of morbillivirus cases that are now easily diagnosed, and that better treatments to prevent outbreaks are currently underway [17,18]. Yet, although morbillivirus infections can now be readily identified, marine mammal stranding events still remain poorly characterized in terms of their etiology [14].

### High throughput sequencing technology for disease identification and surveillance

The use of high throughput sequencing can identify and yield new insights into the virome and microbiome of wildlife [19,20]. This technique does not require prior information about the disease agents and is therefore a promising approach for pathogen identification and surveillance in stranded marine mammals. In this study, we use deep sequencing of cDNA to examine the role of possible pathogenic viruses and bacteria in a stranding event of several harbor seals.

Previous metagenomic studies of marine mammals have focused on the viral and microbial community in the gut, skin, and respiratory tissue [21–23]. As some of the worst marine mammal epidemics have been due to neurotropic diseases (morbillivirus), here, for the first time, we looked at the viral and microbial community present in the brain tissue of harbor seals to identify possible neurotropic pathogens. For this study we sampled seven harbor seals that stranded along California, USA, in the spring of 2009. These animals had abnormalities in the brain that may have been caused by an unknown virus, or an abiotic source. As a comparative group, seven other harbor seals with known causes of death were sampled. We targeted both DNA/RNA viruses to identify the possible viral pathogens in this stranding event. Additionally, we looked at microbial RNA to identify opportunistic or secondary bacterial infections in these animals.

## Material and Methods

### Samples and relevant necropsy information

The National Marine Fisheries (NMFS) authorized the collection of tissue samples from stranded marine mammals and satisfies The Marine Mammal Protection Act (MMPA) regulation 50 CR 216.22 and 216.37.

To evaluate the utility of meta-transcriptomics for analyzing a potential marine mammal neurotropic disease, we acquired 14 harbor seal brain tissue samples that had been previously archived at -80°C. These samples were kindly provided by the Marine Mammal Center (MMC) in Sausalito, CA, USA. Brain tissue samples from stranded animals were either part of the cerebellum or the cerebrum (except UCD7 where the brain tissue type was unknown) and were collected between 2008–2012. These individuals ranged in age from pups (<1 month, n = 3) to weaners (< 1 year, n = 10), and one adult (> 3 years). The dates of stranding, death, and necropsy for each sample are listed in Table 1. Based on veterinarian reports, samples were generally necropsied/sampled a day after death with the exception of com1 and com2 where the date of death was unavailable.

**Table 1:** Summary of necropsy reports and tissue types from harbor seals. PhV-1 = Phocine herpes virus -1. Age range is as follows: Pup < 1month, weaner 1–12 months, and adult > 3 years.

Sample ID	Date of Stranding	Date of Death	Date of Necropsy	Cause of Death	Age	Sex	Tissue
UCD1	4/8/09	7/1/09	7/2/09	Unknown	Weaner	M	Cerebrum back
UCD2	4/9/09	7/26/09	7/29/09	Unknown	Weaner	F	Cerebellum front
UCD3	4/11/09	4/21/09	4/22/09	Unknown	Weaner	M	Cerebrum front
UDC4	4/17/09	7/6/09	7/6/09	Unknown	Weaner	F	Cerebrum front
UCD5	4/20/09	7/12/09	7/13/09	Unknown	Weaner	F	Cerebrum front
UCD6	5/2/09	6/26/09	6/27/09	Unknown	Weaner	M	Cerebrum front
UCD7	6/1/09	7/16/09	7/16/09	Unknown	Weaner	F	Cerebrum front
Comparative1	12/22/08	unknown	12/23/08	Congested blood vessels in the meninges	Adult	F	Cerebrum front
Comparative2	9/7/09	unknown	9/8/09	Extensive parasitism in multiple organs	Weaner	F	Brain tissue unknown
Comparative3	9/17/10	5/2/10	5/3/10	PhV-1	Weaner	M	Cerebrum front
Comparative4	4/28/10	4/28/10	4/30/10	Metabolic abnormalities	Weaner	F	Cerebellum front
Comparative5	3/29/11	4/7/11	4/8/11	Omphalophebitis, bacteria infection, and PhV-1	Pup	M	Cerebrum front
Comparative6	4/16/11	4/24/11	4/25/11	Omphalophebitis	Pup	M	Cerebrum front
Comparative7	5/25/12	5/25/12	5/26/12	Omphalophebitis, septicemia and PhV-1	Pup	F	Cerebrum front/back

Veterinarians and staff at the MMC determined that seven of the stranded harbor seals died from the same yet unknown cause of disease; therefore, we will refer to this subset of animals as “unknown cause of death” (UCD). Brain tissues from UCD samples showed signs of disease including: a viral infection, toxin exposure, hypoxia, or nutrient depletion. According to necropsy reports, due to the coincident timing of animals affected and the lesions in the brain the most parsimonious explanation of this mortality event was a viral agent. Immunochemistry tests were negative for West Nile virus, canine distemper virus, feline coronavirus, and canine parvoviruses, but two UCD samples were PCR positive for phocine herpesvirus-1 (PhV-1). Regardless the veterinarians did not attribute PhV-1 as the cause of death in these animals, as there was no evidence of a herpes viral infection in tissues typically infected by PhV-1. The cause of death varied for the other seven harbor seals that we will refer to here as the “comparative” sample group. Three of the samples from the comparative group were also diagnosed with PhV-1 by PCR. Unlike with the UCD samples, PhV-1 was noted in necropsy reports as a contributing factor in the deaths of the infected comparative animals.

### Meta-transciptome library preparations

At Oregon State University (OSU), brain tissue samples were processed in a biological safety hood and all equipment was cleaned with bleach, ethanol and RNase Away (Thermo Scientific, MA). Gloves were changed before handling a new sample to avoid cross sample contamination. Approximately 0.5ng of frozen brain tissue was removed with a sterile scalpel blade on individual Petri Dishes. Tissue was placed in a 2ml tube and homogenized with a disposable pestle in Trizol (Life Technologies, CA), following the manufacturer's instructions. To remove cellular debris, samples were centrifuged for 10min at 12,000 x g at 4°C. Supernatant was transferred to a new tube and 0.2mL of chloroform, for every 1 mL of Trizol, was added to the supernatant. Samples were centrifuged at 10,000 x g for 18min at 4°C and the aqueous phase transferred for further processing. Equal amounts of 100% ethanol were added to samples and then loaded on an RNeasy kit (Qiagen, CA).

To remove host DNA, 2U of Turbo DNase (Life Technologies, CA) was added to samples for a 9 hr digest. Removal of DNA was visually confirmed by loading 5ul of digest on a gel electrophoresis. Host rRNA was removed using the Ribo-Zero Kit Gold (Human-Mouse-Rat) (Epicentre, WI), following the manufacturer’s directions. RNA was submitted to OSU’s Center for Genome Research and Biocomputing (CGRB) core facility for quality control analysis using the Bioanalyzer 2100 HS- RNA Chip (Agilent technologies, CA). All RNA passed quality control and was converted to cDNA using superscript II Reverse Transcriptase (Life Technologies, CA). Libraries were prepared with the TruSeq paired end cluster kit v.3 (Illumina, CA). Comparative and UCD samples were then sequenced on two lanes of the Hi-Seq 2000 platform from Illumina, with each lane containing a mixture of comparative and UCD samples.

### Bioinformatics

The Illumina output was 100 bps paired-end reads and a final data of ~700 million sequences. The sequence data was quality filtered (phred = 30), trimmed, and adapters and poly A tails removed, using FqTrim [24]. A computational normalization program was used to remove over-represented sequences to provide a more precise method for analyzing lower abundant viral and microbial sequences within the dataset. This program was utilized because the majority of sequence output was to host transcripts, therefore this step eliminated the high coverage harbor seal reads. In turn, this discarded redundant data while decreasing the memory and time needed to run subsequent programs on this large dataset [25].

Host and human DNA were filtered with Bowtie2 [26] by aligning against the Weddell seal genome and the human genome, respectively. Bacteria genomes were identified using BLASTn against the bacterial Refseq database with a minimum *e*-value of 10e-^20^. Sequences not identified as human, host, or bacterial were used for viral annotation. Viral protein similarities were identified using RAPSearch (*e*-value of 10e^-3^) [27] and the viral Refseq protein database with the addition of known marine mammals viruses. Viral taxonomy was identified using NCBI GI and taxonomy ID database, NCBI’s taxonomy tree, and python scripts (S1 Script and S2 Script). To reduce the identification of false positive viral assignments, these sequences were also evaluated using BLASTx to NCBI’s non-redundant database with an *e*-value minimum of 10e^-3^. Potential false positives were also avoided by removing short retrovirus-like similarities (100bp) from the analysis due to the likelihood that they could be host genome retro-elements. Viral families known to be infectious to mammals were further evaluated.

In order to analyze microbiome data, sequences that were quality filtered and digital normalized were used. Bacterial sequences were annotated using BLASTn against the bacterial Refseq database. A stringent *e*-value of 10e^-20^ was used for annotations to reduce ambiguity from short 100bp sequences. Bacterial taxonomy was also identified with S1 and S2 Scripts. Sequences identified as bacterial were used to classify bacteria virulence factors. Here we used the database from Virulence Factors of Pathogenic Bacteria (VFPB) and tblastx with an e-value of 10^−3^ [28].

#### Genome mapping

After quality control with FqTrim, reads were aligned to the Refseq Bacteria database using the program Pathoscope, which incorporates bowtie2 for alignments [29]. Within Pathoscope the option to filter sequences was used to remove DNA alignments to the human and Weddell seal. Sequences from sample UCD6 that aligned to *Coxiella burnetii* were imported into Geneious 9.0.1 Beta [30] to gain information of coverage for each base position across the ~2.0 Mb genome (AE016828.2).

Additionally, coverage information for *C*. *burnetii* RSA 493 plasmid pQpH1 (AE016829.1) was analyzed. After FqTrim, all sequences from sample UCD6 were aligned using bowtie2 to plasmid pQpH1. SAMTools Version: 0.1.19-44428cd [31] was used to obtain coverage information for each base position on the 37Kb plasmid.

#### Phylogenetic analysis

A phylogenetic tree of the harbor seal associated *Coxiella* sp. was constructed using the 16S rRNA gene and the following bacteria (for reference we included the corresponding NCBI accession numbers): *Rickettsia conorii* strain Malish 7 (NR_074480.1), *Rickettsia montanensis* str. OSU 85–930 (NR_074472.1), *Legionella sp*. L-29 gene (AB856218.1) *Legionella hackeliae* strain Lancing 2 (NR_104894.1), *Coxiella burnetii* RSA 493(AE016828.2), *Coxiella sp*. SL 1 (GU797243.1), *Coxiella endosymbiont of Haemaphysalis lagrangei* isolate TSD16 (KC170756.1), and *Coxiella burnetii* Harbor Seal (KT894209). MUSCLE was used to align the 16S rRNA genes [32] and RaXML was used to build a maximum likelihood tree using a 1000 boot strap iterations [33].

### Statistical analyses

Statistical analyses of taxon absolute counts were conducted using the Bioconductor DESeq2 package [34]. DESeq2 uses a negative binomial distribution model and the Wald test for differential expression. Taxa differences were considered significant if the model yielded an FDR adjusted p-value ≤ 0.05. Additional analysis was conducted using Primer6 [35], where absolute taxon counts were Log(x+1) transformed and used for Bray-Curtis measures of similarity. Bray-Curtis values were used to conduct a one-way Analysis of Similarity (ANOSIM) with 999 permutations to compare bacterial community composition between comparative and UCD samples. Also Bray-Curtis values were used for a Similarity Percentages (SIMPER) analysis to determine the contribution of each taxon between and within UCD and comparative samples.

## Results

### Viral consortia in harbor seal strandings

**Fig 1:**
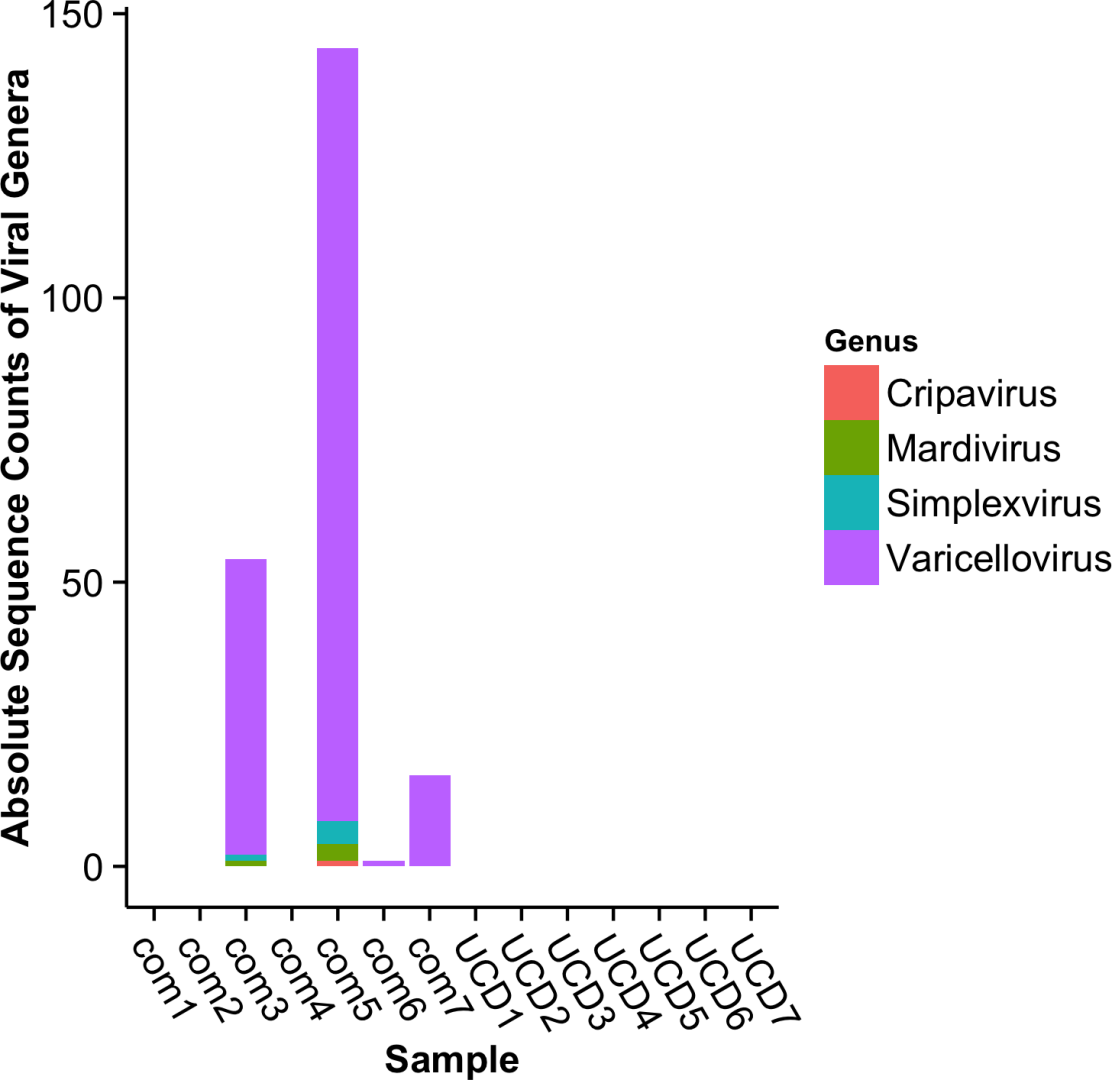
Viral genera associated with stranded harbor seal brain tissues. Absolute sequence counts of assignments to viral genera from comparative (com) and unknown cause of death (UCD) harbor seals.

We aimed to evaluate the microbiome and virome associated with stranded harbor seal brain tissues that displayed signs of a brain disease. A summary of the necropsy reports of each animal sampled in this study are in Table 1 and include: dates of stranding, death, and necropsy, cause of death, age, sex, and tissue type for all fourteen harbor seals. Samples were sequenced on 2 lanes of the Illumina Hi-Seq 2000, yielding ~700 million 100bp paired-end reads. After quality control and the removal of potential host, human, or bacterial sequences, a remaining 13,329,921 sequences were used for viral annotation using RAPSearch and a RefSeq/marine mammal viral database. Identified viral sequences were additionally annotated using BLASTx to NCBI’s non-redundant database. Further only viruses that fell into viral families that are infectious to vertebrates were analyzed. This conservative approach identified a total of 215 reads with identities to known viral sequences of which 100% belonged to the comparative samples (Fig 1).

Interestingly, samples (com3, com5, com7, UCD2, and UCD4) that were previously diagnosed with PhV-1, which belongs to the genus *Varicellovirus*, were indeed found to have sequence assignments to PhV-1 in 3 of the 5 cases (Fig 1). All PhV-1 infected comparative samples (com3, com5, and com7) had sequence annotations to PhV-1, but no UCD animals showed any reads associated with this virus.

#### Distinct microbiomes in UCD harbor seal stranding deaths

**Fig 2:**
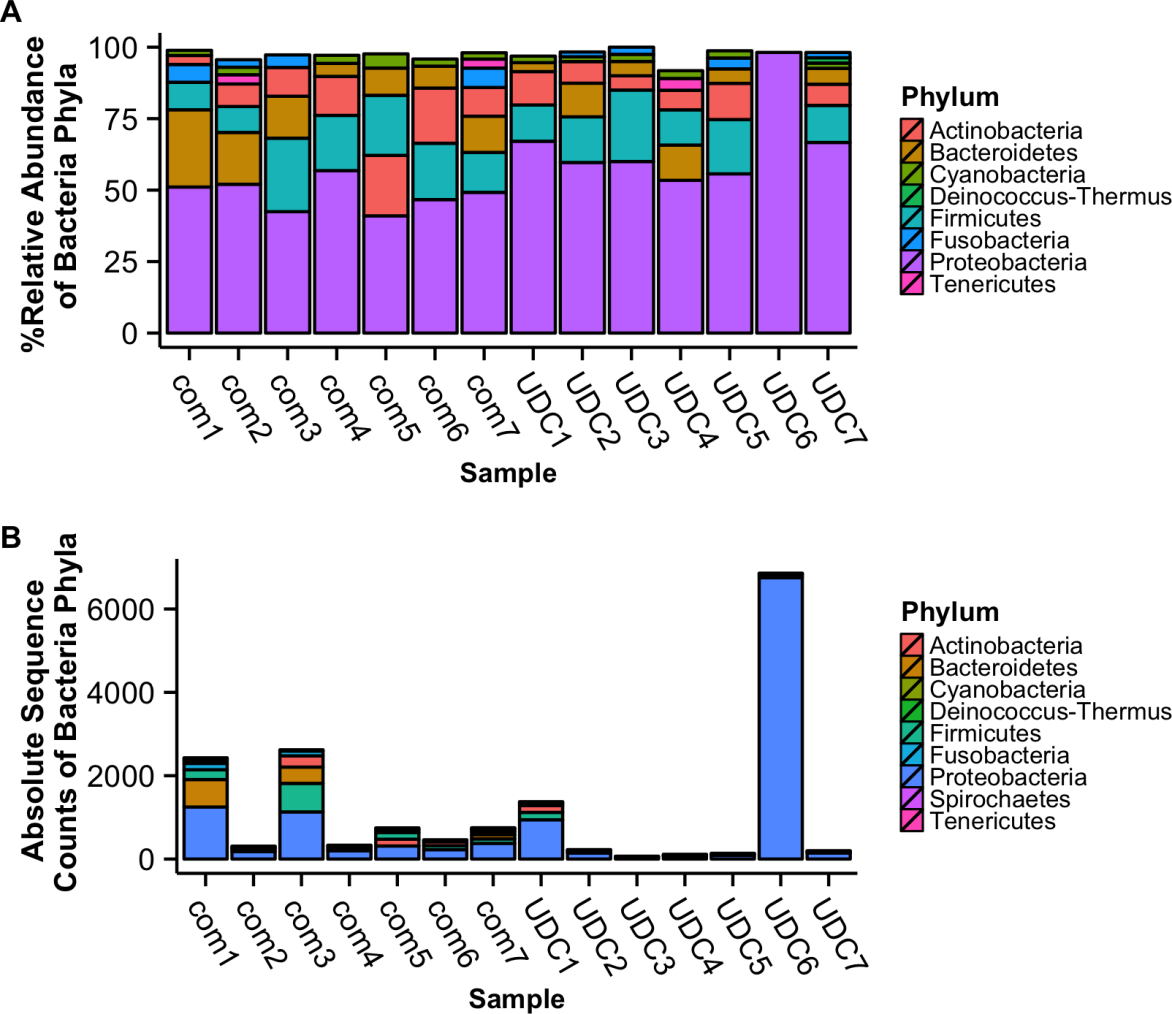
Microbial phyla associated with stranded harbor seal brain tissues. (A) Relative abundance of bacterial phyla with > 1.5% relative abundance samples and (B) bacterial phyla with > 10 absolute sequence counts from comparative (com) and unknown cause of death (UCD).

**Fig 3:**
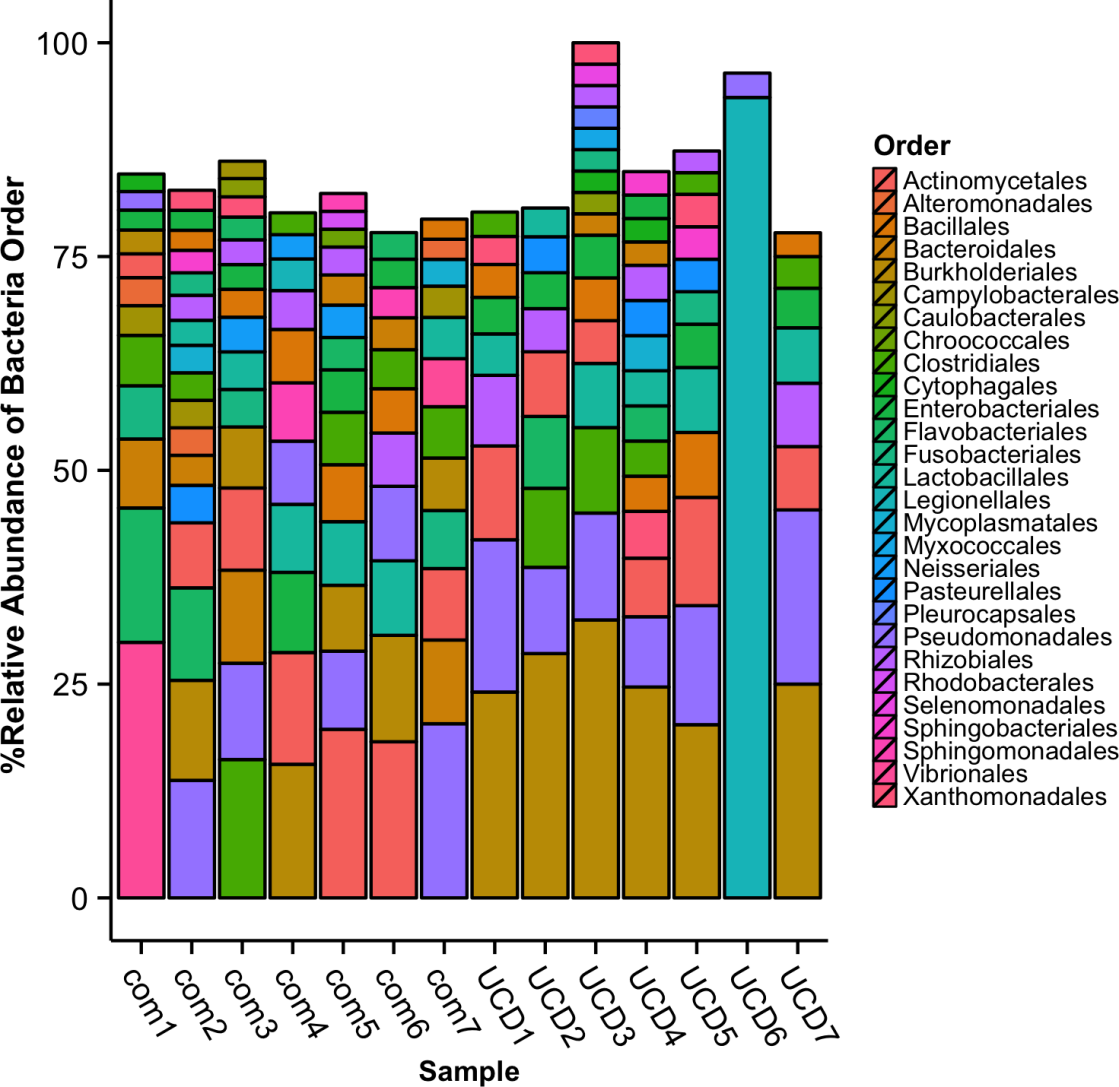
Microbial orders associated with stranded harbor seal brain tissues. Relative abundance of bacterial orders with > 2% relative abundance from comparative (com) and unknown cause of death (UCD) harbor seals.

**Fig 4:**
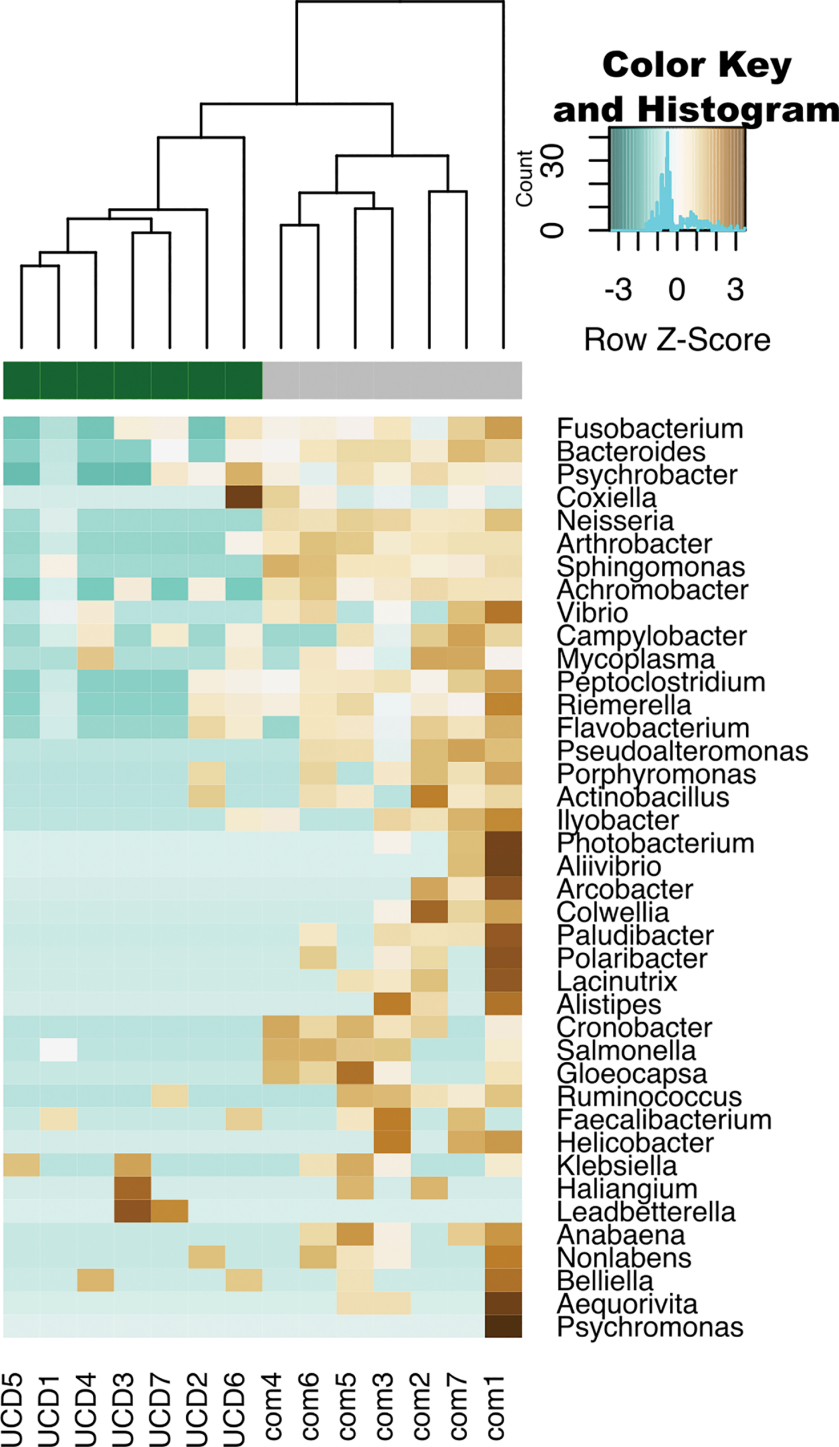
Microbial genera community analysis of brain tissues from comparative (com) and unknown cause of death (UCD) harbor seals. Heatmap hierarchical clustering of the 50 bacterial genera with the highest variance.

After removal of potential host and human sequences the remaining 28,288,156 sequences were used for bacterial annotation. A total of 17,017 sequences were annotated as bacterial; 7,815 reads (0.36%) from the comparative, 9,118 (1.5%) from UCDs, and 85 reads were unable to be assigned taxonomically (Fig 2B). Across all harbor seal brains there were 28 bacteria phyla represented in the sequence data with the dominant taxa similarities to: Proteobacteria, Bacteroidetes, Firmicutes, and Actinobacteria (Fig 2). For bacterial order, Legionellales (93.6%) and Burkholderiales (32.5%) were the most abundant taxa in UCD samples, while Vibrionales (29.9%) and Pseudomonadales (20.4%) were the most abundant in comparative animals (Fig 3). It is noteworthy that com1, the only adult, showed the most disparate microbiome (Fig 4, S1 and S2 Figs). In addition, Bacterial community analysis showed that UCD and comparative samples clustered separately (ANOSIM analysis p = 0.559 and R = 0.4) at the genus level and had a dissimilarity of 69.4% (Fig 4, S1 and S2 Figs, S1 Table).

**Fig 5:**
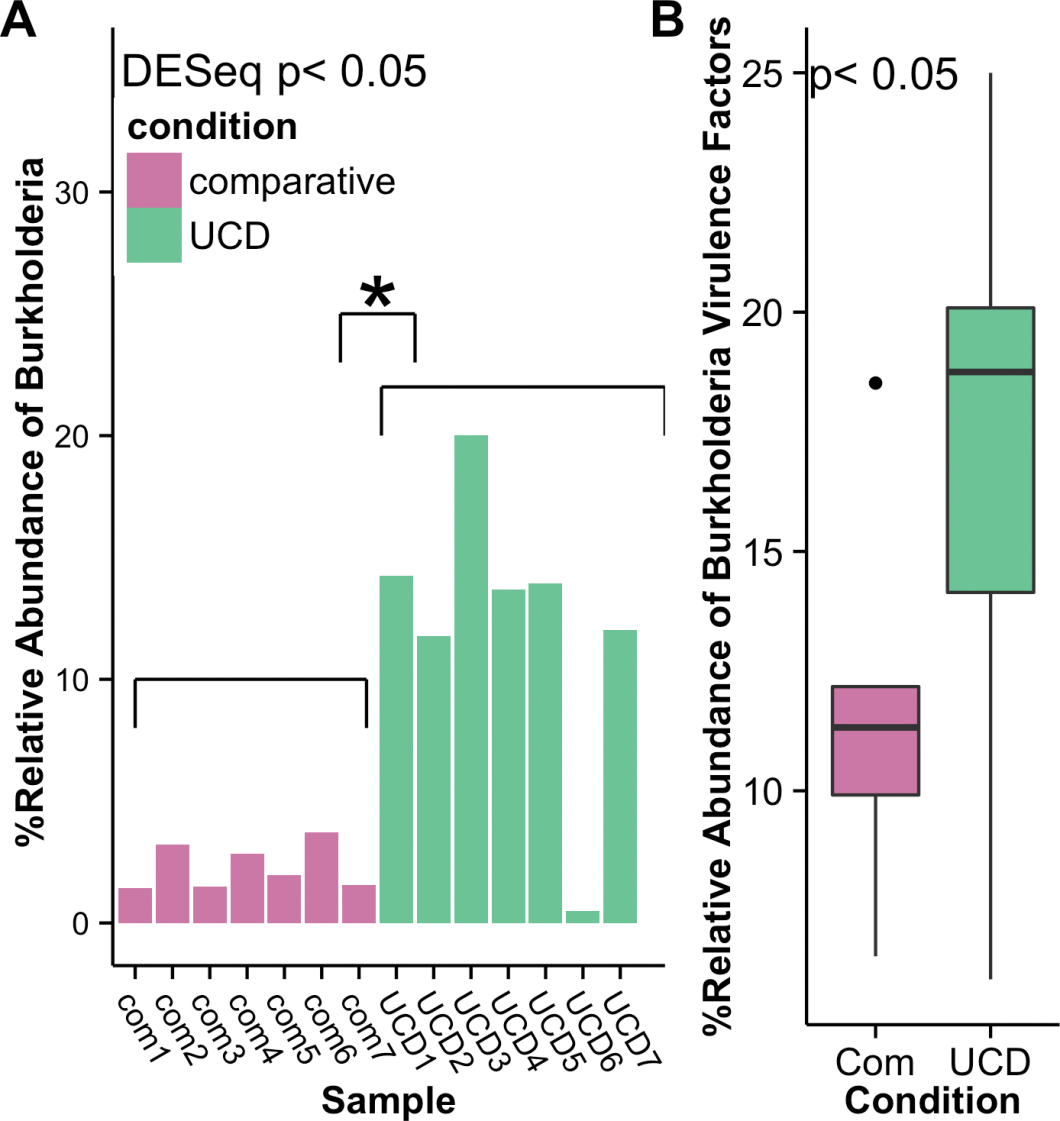
*Burkholderia* and *Burkholderia* virulence factors across samples. (A) Relative abundance of sequences similar to the genera of *Burkholderia* and (B) Relative abundance of sequences similar to *Burkholderia* virulence factors in comparative (com) and unknown cause of death (UCD) harbor seal samples.

From our DESeq2 analysis using absolute taxon counts, we found 50 significantly different bacterial genera between the comparative and UCD samples, with 49 being more abundant in the comparative samples. Only one bacterium, *Burkholderia*, was significantly higher (DESeq padj = 0.032) and had a ~2.8 log2 fold increase in UCD animals (Figs 3 (brown yellow bars plotted at the order level as *Burkholderiales*), & 5A). A SIMPER analysis (S1 Table) also showed that *Burkholderia* had the highest percent contribution (11.4%) to UCD samples. Upon further exploration, the relative abundance of *Burkholderia* virulence factors was also found to be significantly higher in UCD samples then the comparative group (Fig 5B). Sample UCD6 had the lowest relative abundance of *Burkholderia*, but UCD6 also had a unique microbial community with ~94% of its microbiome consisting of *Coxiella burnetii* (order Legionellales) (Figs 3 & 4). This number was confirmed using two bioinformatics pipelines (Pathoscope and BLASTn).

**Fig 6:**
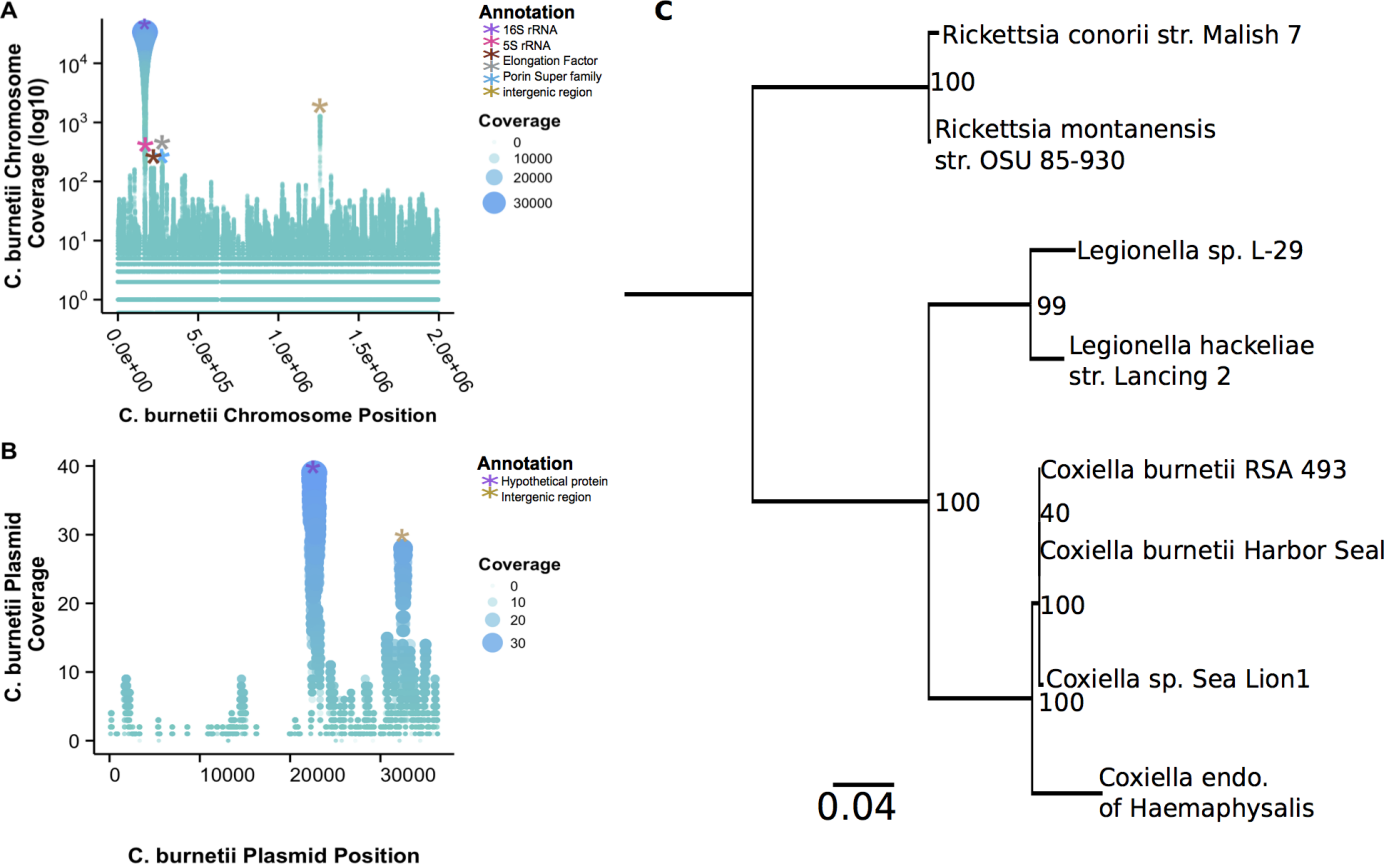
Scatter plot of change in coverage of each base pair position of RNA sequences from sample UCD6 that aligned to (A) *Coxiella burnetii* RSA 493 chromosome and (B) *Coxiella burnetii* RSA 493 plasmid pQpH1. (C) 16S RNA gene maximum likelihood tree with 1000 bootstrap values generated from the following bacterial gene sequences and their corresponding NCBI accession numbers: *Rickettsia conorii* strain Malish 7 (NR_074480.1), *Rickettsia montanensis* str. OSU 85–930 (NR_074472.1), *Legionella sp.* L-29 gene (AB856218.1) *Legionella hackeliae* strain Lancing 2 (NR_104894.1), *Coxiella burnetii* RSA 493(AE016828.2), *Coxiella sp.* SL 1 (GU797243.1), *Coxiella endosymbiont of Haemaphysalis lagrangei* isolate TSD16 (KC170756.1), and *Coxiella burnetii* Harbor Seal (KT894209).

While *Coxiella* was found in 35.7% of the samples it was generally found at less than 4% relative abundance across the communities. However, 319,747 sequences from sample UCD6, spanned across the 2.0Mbp *C*. *burnetii* RSA 493 chromosome and 675 sequences aligned to *C*. *burnetii* RSA 493 plasmid pQpH1 (Fig 6A & 6B). The highest sequence coverages were 6,568X and 1,278X on the chromosome to the 16S rRNA gene and an intergenic region, respectively. The top 6 positions with the highest coverage in the chromosome are listed in Table 2. Region 276,910–277,688 was annotated as unknown in the *C*. *burnetii* chromosome, but upon reannotation it is likely a membrane protein of the porin superfamily. For plasmid pQpHIF, the highest coverage was 39X which codes for a hypothetical protein and at 32X, which was an intergenic region (Fig 6B & Table 2). Furthermore, alignments of the 16S rRNA gene resulted in a 100% sequence identity to *C*. *burnetii* RSA 493 (found in multiple hosts and habitats) and a 99% identity to a *Coxiella sp*. from seal lions (Fig 6C).

**Table 2:** List of the highest coverage regions in *Coxiella burnetii* RSA 493 chromosome and *Coxiella burnetii* RSA 493 plasmid pQpH1 found in sample UCD6.

Annotation	Position on the Genome	Highest X Coverage of Coding Sequence	Genome
16S rRNA	165,579–167,035	33,754	Chromosome
none (intergenic region)	1,259,601–1,259,932	1,278	Chromosome
Membrane protein -Porin superfamily	276,910–277,668	303	Chromosome
5S rRNA	171,960–172,076	279	Chromosome
ompA like transmembrane domain containing protein	273,688–274,363	176	Chromosome
Elongation factor -Tu	223,400–224,593	173	Chromosome
Hypothetical Protein	22,568–23,269	39	Plasmid
none (intergenic region)	32,432–32,567	24	Plasmid

## Discussion

### Harbor seal brain virome

High-throughput sequencing of cDNA and DNA has been used in the past to identify viruses and bacteria associated with their hosts [20,21]. Here we applied this technique to archived marine mammal brain tissues in an attempt to identify the causative agent of a spring 2009 harbor seal stranding event along the Californian coast. The brain tissues of these animals had signs suggestive of an infectious viral or abiotic disease. Yet, although it was originally hypothesized that a viral infection was the culprit of UCD sample deaths, we conclude that this harbor seal stranding event was likely not caused by any known viral agent because UCD animals had no viral read similarities. Alternatively, the viral type that caused the death of these animals could be divergent enough that the viral database used for annotation could not recognize this virus. Moreover, it may be possible that sequencing was either not deep enough to detect certain viral genomes or transcripts within the background of the host and bacterial reads or that rRNA removal methods eliminated important viral sequences as reported in [36]. However, other studies report an increase in viral reads with the use of rRNA removal for viral enrichment [37]. Regardless, other viral enrichment methods could potentially eliminate these combined issues [38]. Lastly, it is a possibility that due to late stage of the disease, the viral pathogen was either no longer present in the tissue samples or at a stage in its lifecycle where transcription was concluded and therefore undetectable using these methods.

Of note, we standardized our viral analysis by using animals that the Marine Mammal Center found to be PCR positive for Phocine herpes-virus (PhV-1). Our deep sequencing methods, detected PhV-1 in PhV-1 infected comparative samples, but not in PhV-1 infected UCD samples. Unlike comparative samples, veterinarians did not attribute PhV-1 to cause disease in UCD animals because tissues had no signs of a herpes viral infection and PhV-1 was not found in other tissues. Thus this may imply that PCR results in UCD samples may have been PCR positive due to the presence of a latent viral infection where transcripts are present but at very low abundances [39,40] and thus were not captured with our methods. Therefore, sequencing of cDNA may be more effective at identifying active viral (versus latent) infections, but further studies need to be conducted.

### Harbor seal brain microbiome

Across samples, annotated bacterial transcripts were mostly similar to the phylum Proteobacteria (70.1%). Our findings are in line with previous brain metagenomes that reported Proteobacteria to be the most abundant phylum in human and nonhuman primate brains regardless of health state [41]. In our study, we found that the only adult in our analysis had the most disparate microbial community. This might have been due to the age of the animal or a difference in disease microbial community progression. It would be interesting to evaluate metagenomes of additional adults to determine if adult harbor seals have different brain microbiomes from their pup/weaning counterparts.

Furthermore, out of the 50 significantly differentiated bacterial taxa, we found that only *Burkholderia* cDNAs were significantly higher in UCD samples. The importance of *Burkholderia* in UCD samples was also apparent by the high prevalence of this genus, and the relatively higher *Burkholderia* specific virulence factors in UCD animals. Therefore, this may be indicative of an opportunistic neurotropic pathogen common in the 2009 harbor seal pup-stranding event.

*Burkholderia* is a genus that is ubiquitous and inhabits various niches. This bacterium has been isolated in marine mammal brains, but infections have mostly been reported in captive marine animals in Southeast Asia [42]. To our knowledge this is the first report of a wild harbor seal *Burkholderia* infection in the Americas. *Burkholderia* is known to cause zoonotic diseases, such as melioidosis that leads to abscesses, and typically affects human populations in South Asia and Northern Australia. Incidence of this particular disease is increasing in areas such as Northeast Thailand, but is not typically seen in the United States [43]. Given the increase of human-marine mammal interactions [6] and high *Burkholderia* prevalence in our samples (100%), marine mammals may be a source for *Burkholderia* zoonoses.

Although *Burkholderia* was found in relatively high abundances in most UCD samples it was least abundant in UCD6. This is probably due to the distinct microbial community found in UCD6, with *Coxiella burnetii* dominating the microbiome by ~ 94%. Of the four other samples that had *Coxiella* sequences, *Coxiella* represents only < 4% of their microbiome. Interestingly, *C*. *burnetii* antibodies have been found in 34% of healthy harbor seals in the Pacific Northwest of the USA [44]. In our sample size of 14 harbor seals, we were able to identify a similar prevalence of *Coxiella* (35.7%). This finding suggests that deep sequencing may be a valid method for identifying the prevalence of microbes in archived marine mammal tissues.

*C*. *burnetii* is the causative agent of Query (Q) fever, considered a ubiquitous zoonotic disease, and in marine mammals a known cause of placentitis [44–46]. Since sample UCD6 was a weaner, the source of infection may have been from the mother’s placenta; therefore, we hypothesize that *C*. *burnetii* played an important role in the death of UCD6 and may be indicative of an opportunistic neurotropic pathogen that was transmitted through the placenta. As this is the first report of *C*. *burnetii* infecting marine mammal brains; it will be important to research the effects of this bacterium on the brain of developing harbor seals.

Also, we found that *C*. *burnetii* from this study was more closely related to strain RSA23, but still similar to the sea lion strain (99% identity). Given the high abundance of this bacterium in the brain of UCD6 and the relatedness of the strains, future studies should evaluate the prevalence and rate of *C*. *burnetii* in the brains of other stranded marine mammals.

Additionally, our data show that *C*. *burnetii* in UCD6 had the highest expression rate at the 16S rRNA gene with a 33,754X coverage, which is an expected result since rRNA can account for 95–98% of total RNA in bacteria. The second most abundant region had a 1,276X coverage and had no assignment in the BLAST database. Due to its high abundance and lack of annotation, we hypothesize that this may be a regulatory RNA produced during active infections, and we plan to investigate this in the future study. Alternatively, this can potentially be *C*. *burnetii* DNA contamination that was not eliminated during the DNase procedure, although, this hypothesis seems unlikely due to the extensive 9 hr. DNase treatment and because the sample was verified to contain no genomic DNA using gel electrophoresis.

Furthermore, OmpA and elongation factor–Tu were some of the most highly expressed transcripts in this data at 176X and 173X coverage, respectively. OmpA is an important virulence factor of *C*. *burnetii* needed for invasion of host cells [47] and elongation factor–Tu is a core translational gene. Therefore, these data indicate that this was an active infection where *C*. *burnetii* was invading and replicating in UCD6 brain tissues. In addition, our results show that there were two highly abundant regions on *C*. *burnetii* plasmid pQpH1 expressed in this brain tissue. One region annotated to a hypothetical protein and the other to an intergenic region of the plasmid. The high abundance of these regions imply that they may be involved during infection.

## Conclusions

Deep sequencing of cDNA proved to be an informative tool in detecting viral and bacterial pathogens in harbor seal brains and therefore should be useful for future surveillance of zoonotic pathogens in wildlife. Although the cause of death of the seven stranded seal pups from 2009 is still unknown, we concluded that a viral pathogen was not the likely cause of death in these animals, and that two bacterial pathogens, *Burkholderia* and *Coxiella burnetii*, may be involved in this stranding event. It is key to note that while our methods were able to detect various bacterial signatures, bacterial pathogens likely did not cause the death of these animals. This is because necropsy reports did not note any signs or brain lesions from bacteria as the cause death. Therefore, significant bacteria pathogens detected in this study such as *Burkholderia* and *C*. *burnetii* are more likely opportunistic pathogens in these animals. Future work will focus on the transcriptome of these individuals to try to identify if an abiotic source contributed to the deaths of these animals.

## Supporting Information

S1 Fig Microbial genera level community clusters analysis of the brain tissue of harbor seal samples. Principal Coordinate Analysis (PCA) of bacterial community of com (comparative) and UCD (unknown cause of death) harbor seals.
(TIFF)

S2 Fig Microbial genera level community analysis of brain tissues from comparative (com) and unknown cause of death (UCD) harbor seals. Heatmap hierarchical clustering of the 30 most abundant bacterial genera.
(TIFF)

S1 Script my_gi_to_taxid.py to get taxIDs from GI numbers. The input is a list of sequence identifiers (column1) and their corresponding GI numbers (column2). The output can be used to run S2 script (my_taxid_to_taxon.py).
(PY) Click here for additional data file.

S2 Script my_taxid_to_taxon.py retrieves NCBI taxonomy lineage from taxIDs. The input is a list of sequence identifiers (column1) and their corresponding taxIDs numbers (column2).
(PY) Click here for additional data file.

S1 Table SIMPER analysis output of the top 20 bacterial genera similarities within and between com (comparative) and UCD (unknown cause of death) harbor seals. (PNG)
