# Correction: Brain Meta-Transcriptomics from Harbor Seals to Infer the Role of the Microbiome and Virome in a Stranding Event

**DOI:** 10.1371/journal.pone.0146208

**Published:** 2015-12-29

**Authors:** Stephanie M. Rosales, Rebecca Vega Thurber

In Table 1, the Date of Stranding for Comparative3 is incorrect. Please see the corrected Table 1 here.

**Table 1:** Summary of necropsy reports and tissue types from harbor seals. PhV-1 = Phocine herpes virus -1. Age range is as follows: Pup < 1month, weaner 1–12 months, and adult > 3 years.

Sample ID	Date of Stranding	Date of Death	Date of Necropsy	Cause of Death	Age	Sex	Tissue
UCD1	4/8/09	7/1/09	7/2/09	Unknown	Weaner	M	Cerebrum back
UCD2	4/9/09	7/26/09	7/29/09	Unknown	Weaner	F	Cerebellum front
UCD3	4/11/09	4/21/09	4/22/09	Unknown	Weaner	M	Cerebrum front
UDC4	4/17/09	7/6/09	7/6/09	Unknown	Weaner	F	Cerebrum front
UCD5	4/20/09	7/12/09	7/13/09	Unknown	Weaner	F	Cerebrum front
UCD6	5/2/09	6/26/09	6/27/09	Unknown	Weaner	M	Cerebrum front
UCD7	6/1/09	7/16/09	7/16/09	Unknown	Weaner	F	Cerebrum front
Comparative1	12/22/08	unknown	12/23/08	Congested blood vessels in the meninges	Adult	F	Cerebrum front
Comparative2	9/7/09	unknown	9/8/09	Extensive parasitism in multiple organs	Weaner	F	Brain tissue unknown
Comparative3	3/14/10	5/2/10	5/3/10	PhV-1	Weaner	M	Cerebrum front
Comparative4	4/28/10	4/28/10	4/30/10	Metabolic abnormalities	Weaner	F	Cerebellum front
Comparative5	3/29/11	4/7/11	4/8/11	Omphalophebitis, bacteria infection, and PhV-1	Pup	M	Cerebrum front
Comparative6	4/16/11	4/24/11	4/25/11	Omphalophebitis	Pup	M	Cerebrum front
Comparative7	5/25/12	5/25/12	5/26/12	Omphalophebitis, septicemia and PhV-1	Pup	F	Cerebrum front/back
