# Supplementary figures and images for: Brain Meta-Transcriptomics from Harbor Seals to Infer the Role of the Microbiome and Virome in a Stranding Event
Source: PLoS One. 2015 Dec 2;10(12):e0143944. doi: 10.1371/journal.pone.0143944 (PMC4668051; doi:10.1371/journal.pone.0143944)

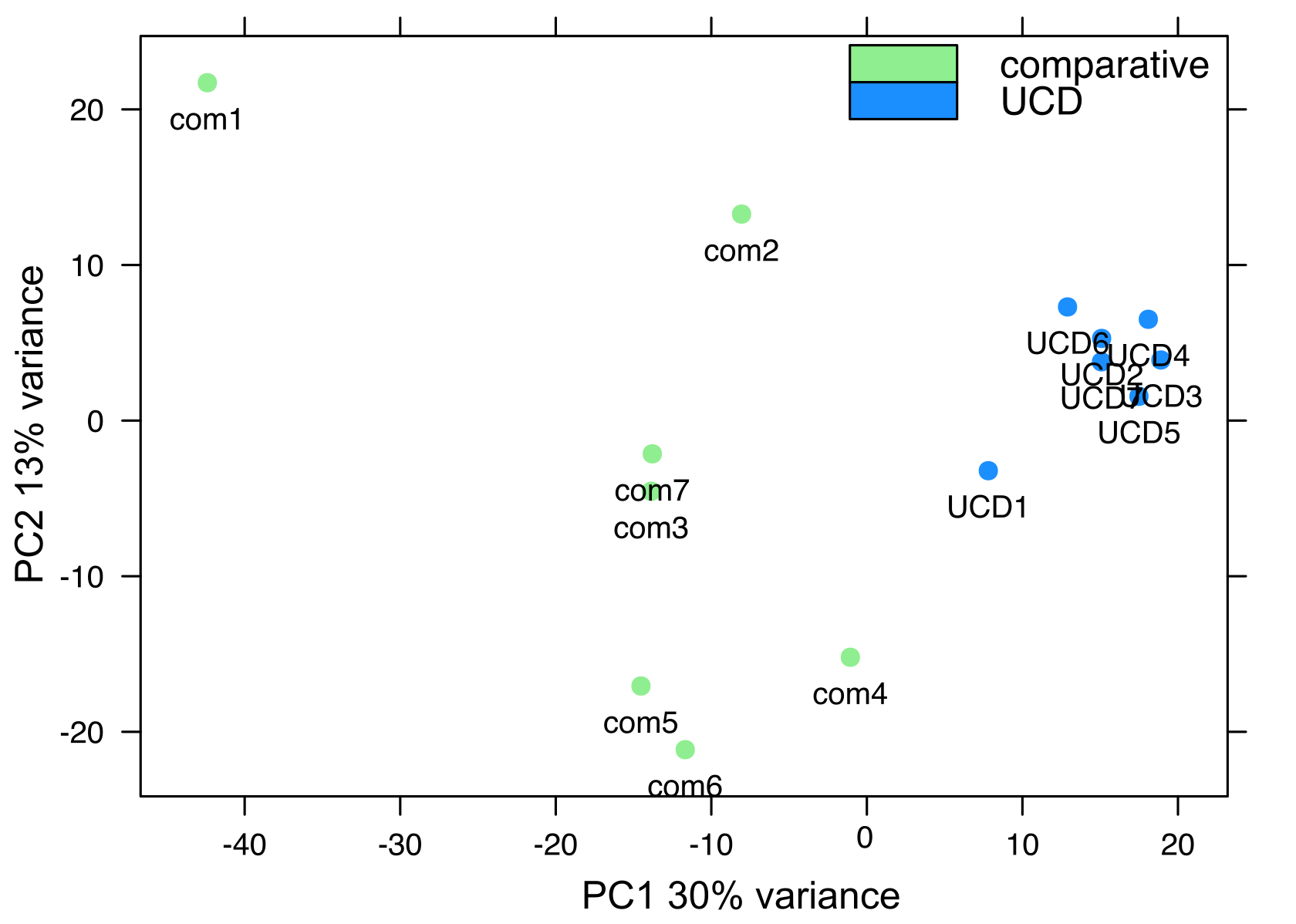

Supplement: S1 Fig — Principal Coordinate Analysis (PCA) of bacterial community of com (comparative) and UCD (unknown cause of death) harbor seals. (TIFF) [file pone.0143944.s001.tiff]

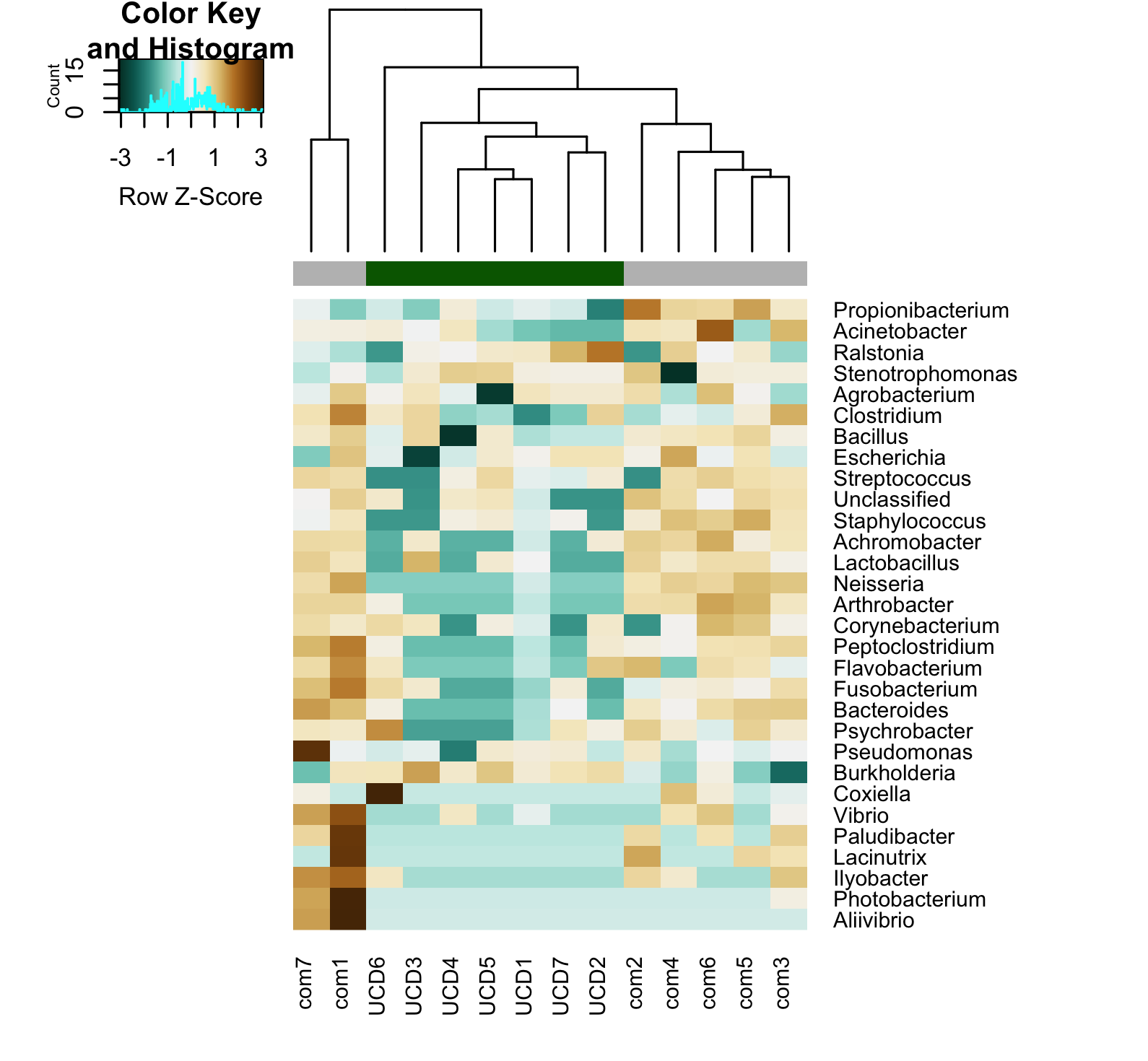

Supplement: S2 Fig — Heatmap hierarchical clustering of the 30 most abundant bacterial genera. (TIFF) [file pone.0143944.s002.tiff]

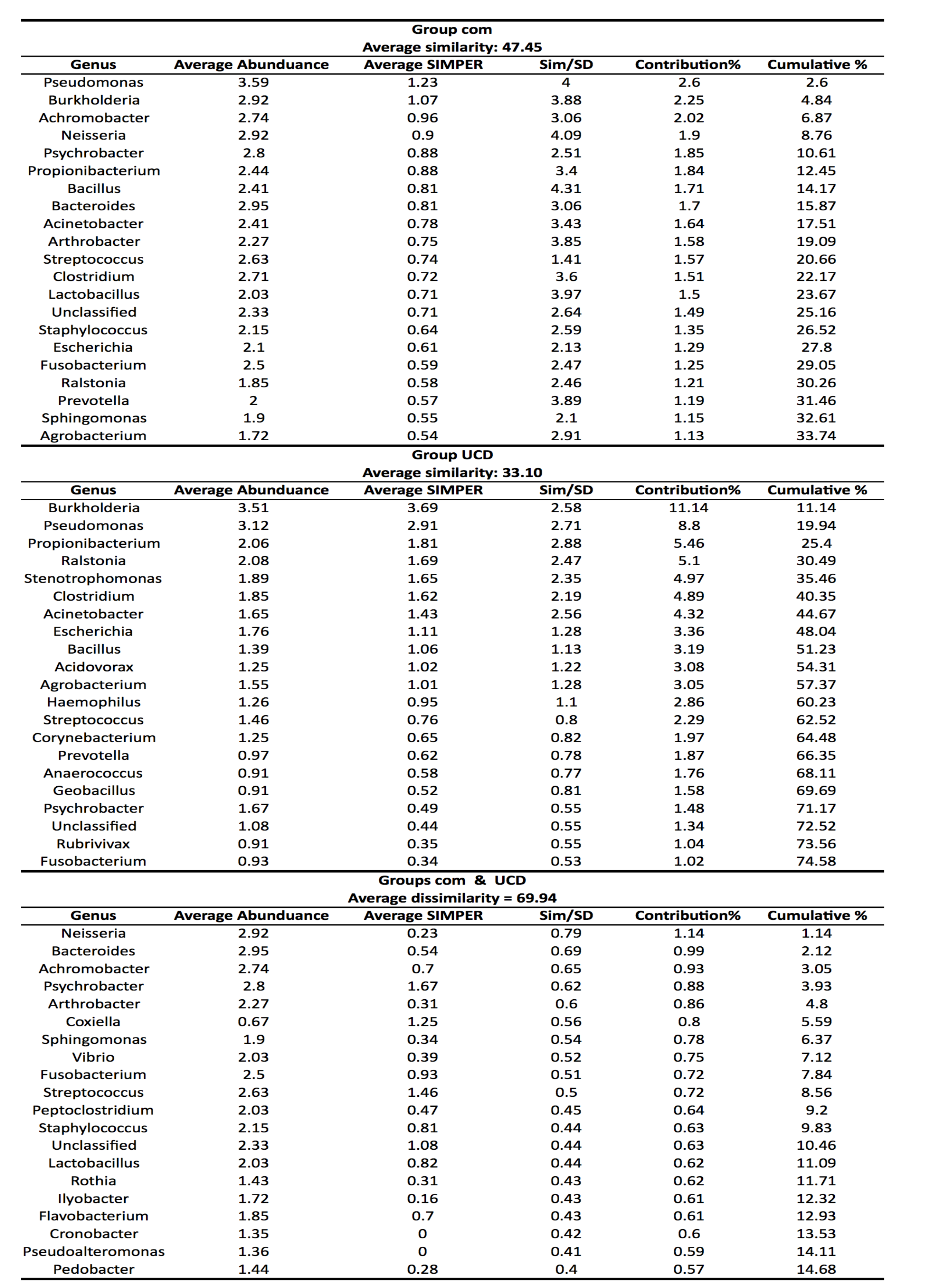

Supplement: S1 Table — (PNG) [file pone.0143944.s005.png]
